# Sex-specific non-linear associations between body mass index and impaired pulmonary ventilation function in a community-based population: Longgang COPD study

**DOI:** 10.3389/fphar.2023.1103573

**Published:** 2023-03-09

**Authors:** Hao Huang, Xueliang Huang, Jiaman Liao, Yushao Li, Yaoting Su, Yaxian Meng, Yiqiang Zhan

**Affiliations:** ^1^ Department of Epidemiology, School of Public Health (Shenzhen), Sun Yat-Sen University, Shenzhen, China; ^2^ Nanlian Community Health Service Center, Shenzhen Longgang Central Hospital, Shenzhen, China

**Keywords:** body mass index, COPD, Epidemiology, lung function, sex

## Abstract

**Aim:** To investigate the prevalence of pulmonary airflow limitation and its association with body mass index (BMI) in a community-based population in Shenzhen, China.

**Methods:** Study participants were recruited from Nanlian Community in Shenzhen, China, and spirometry was performed to assess lung function including forced vital capacity (FVC), forced expiratory volume in 1 s (FEV_1_), FEV_1_/FVC ratio, and FEV_1_ divided by predicted value. Pulmonary airflow limitation was determined by the Chinese Guideline of Pulmonary Function Examination. Multivariable logistic regression models were used to examine the association between BMI and pulmonary airflow limitation. Age, sex, educational attainment, occupation, and current cigarette smoking were used as potential confounders.

**Results:** Of the 1206 participants, 612 (50.7%) were men and 594 (49.3%) were women with the average age being 53.7 years old. After adjusting for age, sex, educational attainment, occupation, and current cigarette smoking, higher BMI was associated with lower odds (odds ratio: 0.98, 95% confidence interval: 0.97, 0.99) of pulmonary airflow limitation by assuming a linear relationship. Further investigation of the interaction terms, we found that the magnitudes of the associations differed in men and women. A U-shaped relationship was observed in women, while the association was almost linear in men.

**Conclusion:** The relationship between BMI and pulmonary airflow limitation was U-shaped in women and linear in men.

## Introduction

Chronic obstructive pulmonary disease (COPD) is one of the most common respiratory diseases including emphysema and chronic bronchitis (Safiri et al., 2022). The prevalence of COPD was estimated to be 10.3% among people aged 30–79 years (Adeloye et al., 2022). It is characterized by irreversible airflow blockage and long-term respiratory symptoms. It progresses slowly with daily activity becoming harder. While COPD is difficult to cure and recover, it is treatable and modifiable from the public health perspective.

The causes of COPD pathogenesis are thought to be long-term exposures to harmful particles that can irritate the lung and lead to inflammation. For example, the most significant and outstanding risk factor is tobacco smoking which is estimated to contribute to almost half of COPD (Hikichi et al., 2019; Adeloye et al., 2022). Other risk factors include pollution, older age, being man, metabolic profiles, and genetics (Hooper et al., 2012; Sakornsakolpat et al., 2019; Silverman, 2020). While the disease burden of COPD can be reduced by mitigating exposures to smoking and pollution, the role of obesity in COPD pathogenesis is largely controversial. A recent meta-analysis and systematic review summarizing previous results found that being underweight was associated with higher risks of COPD and being overweight was associated with lower risks (Zhang et al., 2021). While the relationships between obesity and COPD reported in these studies were assumed to be linear, they were seldom tested in the analyses (Melo et al., 2014; Tang et al., 2022). Indeed, one study found that the relationship between obesity and COPD seemed to be U-shaped in Canadian (Chen, 2000) and British cohorts (Garcia Rodriguez et al., 2009). Further investigations of this relationship in other ethnic populations are warranted.

In the present study, we aimed to revisit the relationship between obesity, as measured by body mass index (BMI), and impaired pulmonary ventilation function in a community-based population in Shenzhen, China. Further, we sought to examine if there were interactions between BMI and gender. We made the hypothesis that a higher BMI was related to lower risks of impaired pulmonary ventilation function and gender could modify the observed associations.

## Methods

### Study population

Study participants were recruited from local residential communities using a convenience sampling method in Longgang District, Shenzhen, China. The primary aim of this survey was to investigate the prevalence of COPD and related risk factors as well as long-term health outcomes in this population. Citizens or local permanent residents (people who are registered as Shenzhen citizens, but not those who lived outside of Shenzhen for no less than 6 months, and non-registered Shenzhen citizens who have temporary residence permits and have lived in Shenzhen for no less than 6 months) who were 18 years or older were recruited. Data collection was carried out from Jan to December 2021. At the beginning of the survey, our administrative staff collaborated with local administrative heads and told them our aim and methods of this survey. Because of their collaboration, we could share and inform them of our study design through social media and printed handouts. All families in the communities were informed and only one member of a house was invited to our study. An onsite questionnaire was administered by trained staff members and health professionals at the corresponding community health service centers. In total, 1206 participants aged 18 years and over completed the questionnaires.

### Spirometry

Trained health professionals performed spirometry using a U-Breath PF680 spirometer (Wang et al., 2022) (e-LinkCare Meditech Co., Ltd. Zhejiang, China). Study participants were instructed to lift their chins, lengthen their necks slightly, and place a nose clip on their noses during testing in order to prevent air leaks. They were asked to practice these procedures twice before carrying out a couple of formal maximal forced expiratory tests. In each maneuver, the participant took the deepest breath possible to fill the lungs with air, then put the mouthpiece into his/her mouth making a tight seal, and then blew the air out as hard and fast as possible.

Forced vital capacity (FVC), the first second of forced expiration volume (FEV_1_), FEV_1_/FVC ratio, percentage of predicted FEV1 value (FEV_1_%predicted), and lower limit of normal (LLN) were recorded. According to the Chinese Guideline of Pulmonary Function Examination (Asthma, 2013), pulmonary airflow limitation was graded as any one of the following: minor (FEV_1_%predicted ≥70%, but < LLN or FEV_1_/FVC ratio < LLN), moderate (FEV_1_%predicted: 60%–69%), moderate-severe (FEV_1_%predicted: 50%–59%), severe (FEV_1_%predicted: 35%–49%), and extremely severe (FEV_1_%predicted <35%).

COPD was defined as FEV1/FVC <0.7 and was graded as mild, moderate, severe, and very severe according to The Global Initiative for Chronic Obstructive Lung Disease (GOLD) system: GOLD 1 - mild: FEV1 ≥80% predicted; GOLD 2 - moderate: 50% ≤ FEV1 <80% predicted; GOLD 3 - severe: 30% ≤ FEV1 <50% predicted; GOLD 4 - very severe: FEV1 <30% predicted.

### Potential confounders

Weight and height were measured by trained staff using a calibrated instrument. Height was recorded to the nearest 0.5 cm wearing no foot wares, and weight was assessed to the nearest 0.1 kg. Body mass index (BMI) was then estimated as weight (kg) divided by the square of height (m). Educational attainment was recorded as primary school and below, middle school, and college and above. Cigarette smoking was assessed as current smoking and non-smoking. Comorbidity was defined as any of the following diseases: heart failure, chronic pulmonary heart disease, diabetes, or respiratory failure.

### Statistical analysis

Descriptive statistics were presented in men and women separately. Mean and standard deviations were presented for continuous variables, while counts and percentages (%) were calculated for categorical phenotypes. Multivariable logistic regression models were employed to estimate the associations and magnitudes between BMI and the risks of pulmonary airflow limitation. Results are described as odds ratio (OR) and 95% confidence intervals (CIs) after controlling for potential confounders. A potential non-linear relationship between BMI and pulmonary airflow limitation was tested and investigated by treating BMI as restricted cubic splines. We further carried out gender-specific analyses to test the potential effect modification of sex. These analyses were also conducted for COPD. All the analyses were two-tailed and *p* < 0.05 was taken as statistically significant. All analyses were performed using R 4.1.

## Results

### Demographic Characteristics of the study population

Among the 1206 participants, 612 (50.7%) were men and 594 (49.2%) were women with the average age being 53.7 (standard deviation: 9.0) years old as shown in Table 1. The prevalence of current cigarette smoking was 31% while more than half of the participants (52.0%) had a BMI over 24 kg/m^2^ and only 10.8% of the participants had a college degree and above.

**TABLE 1:** Basic demographic characteristics of study population in longgang COPD study, shenzhen, China, Jan–December 2021.

Variables		N	Pulmonary airflow limitation, n (%)
Gender	Men	612	259 (42.3)
	Women	594	159 (26.8)
Age Groups	18–44	206	70 (34.0)
	45–59	686	218 (31.8)
	60-	314	130 (41.4)
BMI (kg/m^2^)	<18.5	35	17 (48.6)
	18.5–24	544	209 (38.4)
	>24	627	192 (30.6)
Educational Attainment	College and above	130	41 (31.5)
	Middle School	773	269 (34.8)
	Primary School and below	303	108 (35.6)
Current Cigarette Smoking	Yes	375	184 (49.1)
	No	831	234 (28.2)
Comorbidity	Yes	276	140 (64.8)
	No	930	278 (30.0)

BMI: body mass index.

### Associations between body mass index and pulmonary airflow limitation

The prevalence of pulmonary airflow limitation was 34.7% (minor: 27.9%, moderate: 3.9%, moderate-severe: 1.2%, severe: 1.0%, and extremely severe: 0.7%). Multivariable logistic regression analysis revealed that higher BMI was associated with lower risks of pulmonary airflow limitation with the OR (95% CI) being 0.95 (0.90, 0.97) per one unit increment in BMI when assuming a linear relationship and adjusting for age, sex, and current cigarette smoking. Compared with people with a normal BMI (18.5–24 kg/m^2^), those with a BMI <18.5 had a higher risk of pulmonary airflow limitation (OR:1.41, 95% CI: 0.68, 2.92), and those with a BMI >24 had a lower risk of pulmonary airflow limitation (OR: 0.63, 95% CI: 0.46, 0.87) when categorizing BMI into three groups. Further adjusting for educational attainment yielded comparable results (Table 2).

**TABLE 2:** Association between body mass index and pulmonary airflow limitation in Longgang COPD Study, Jan-December 2021, Shenzhen, China, OR (95% CI).

BMI	Model 1	Model 2
<18.5	1.41 (0.68, 2.92)	1.42 (0.70, 2.89)
18.5–24	Reference	Reference
>24	0.63 (0.46, 0.87)	0.61 (0.47, 0.79)
Continuous, 1 kg/m^2^ increment	0.95 (0.90, 0.97)	0.98 (0.97, 0.99)

Model 1: age, gender, current cigarette smoking; Model 2: age, sex, current cigarette smoking, and educational attainment. OR, odds ratio; CI: confidence interval.

**FIGURE 1:**
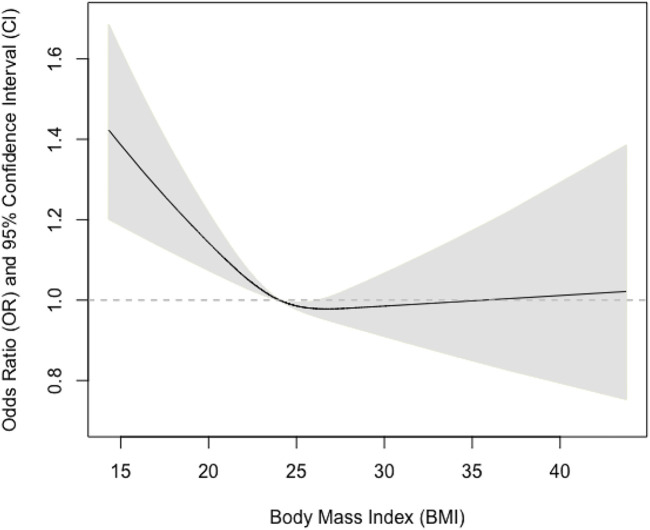
Association between body mass index and pulmonary airflow limitation in men and women.

**FIGURE 2:**
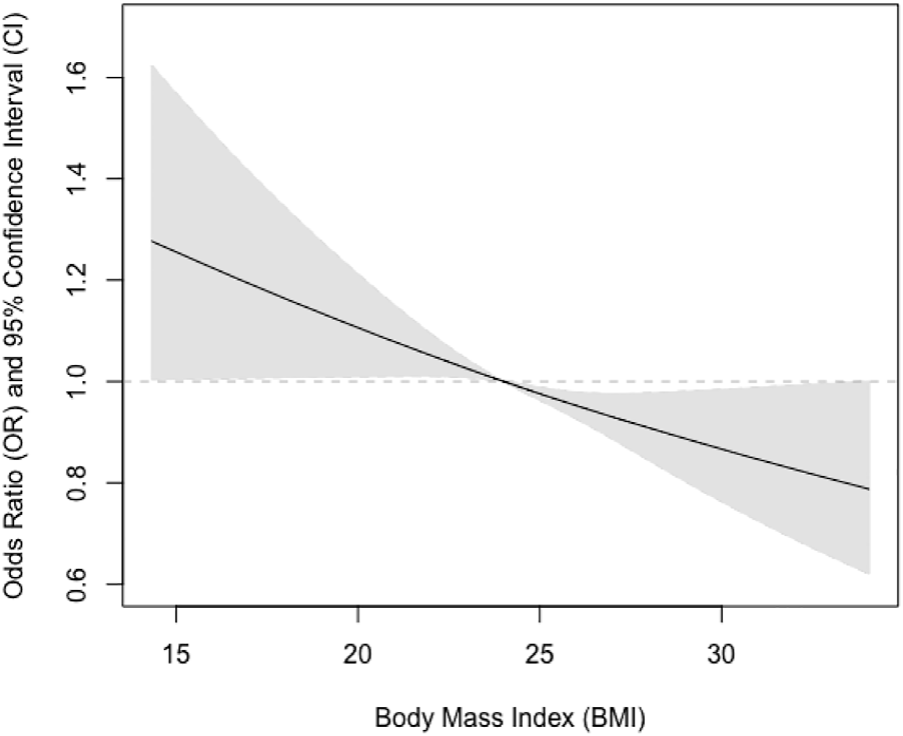
Association between body mass index and pulmonary airflow limitation in men.

**FIGURE 3:**
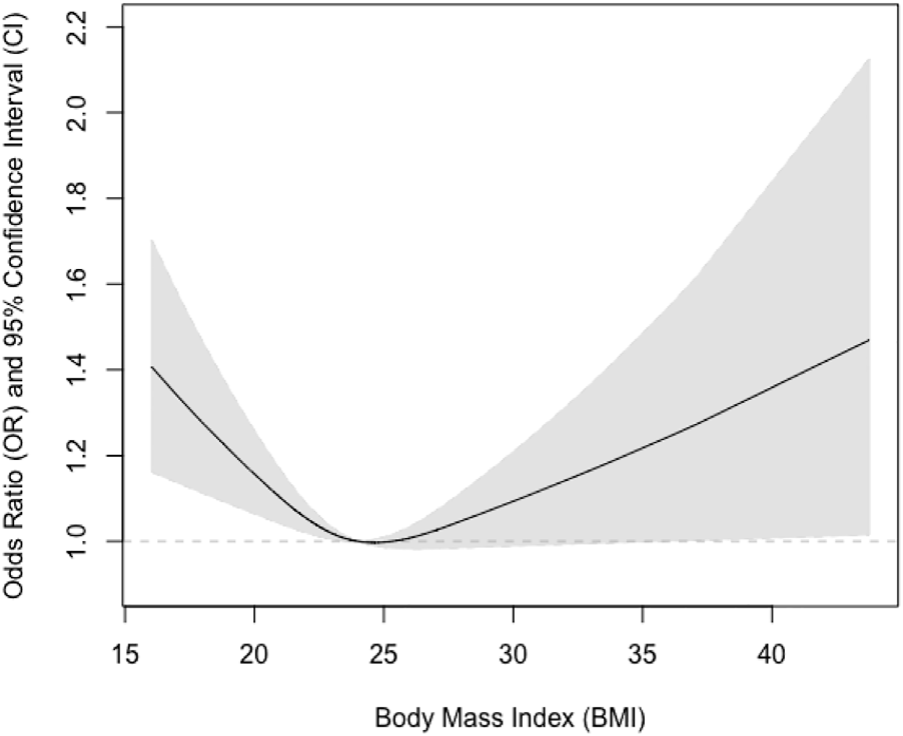
Association between body mass index and pulmonary airflow limitation in women.

We further tested if sex could modify the observed associations and found that the interaction term between sex and BMI was statistically significant (*p* = 0.03). We also examined if there were non-linear associations between BMI and pulmonary airflow limitation using restricted cubic splines (Figure 1). We further found that the relationship was almost linear in men (Figure 2), while a U-shaped relationship was observed in women (Figure 3).

**FIGURE 4:**
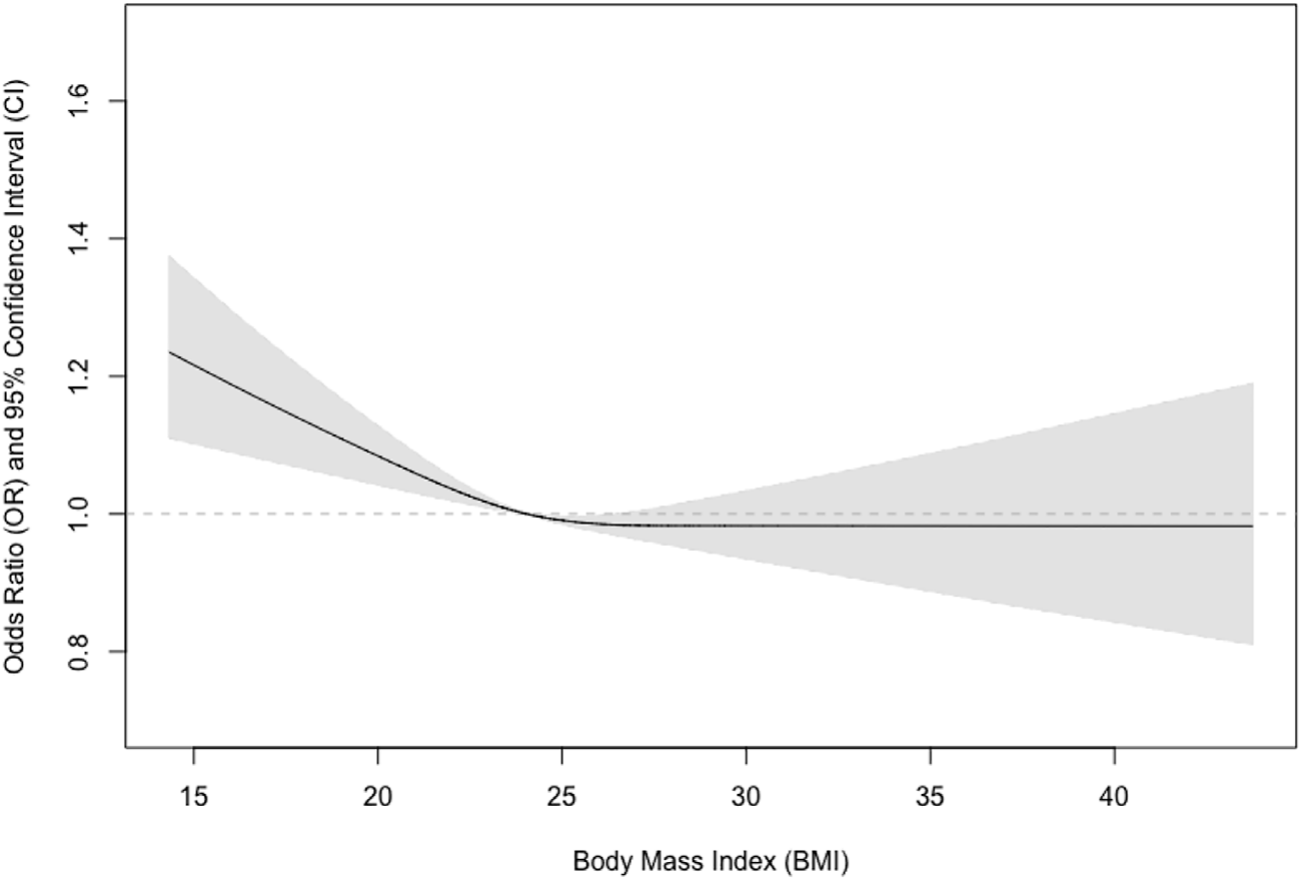
Association between body mass index and COPD in men and women.

**FIGURE 5:**
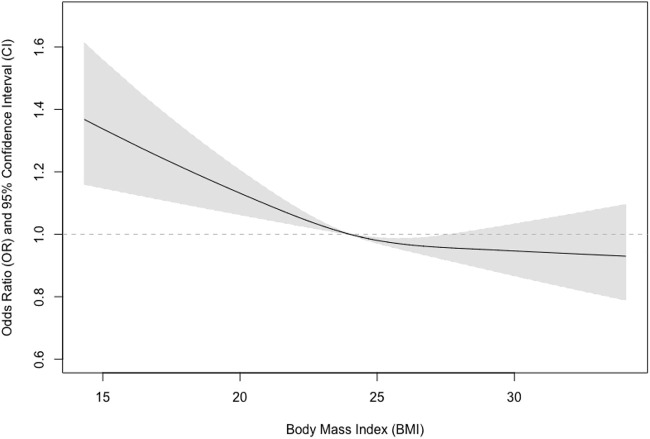
Association between body mass index and COPD in men.

**FIGURE 6:**
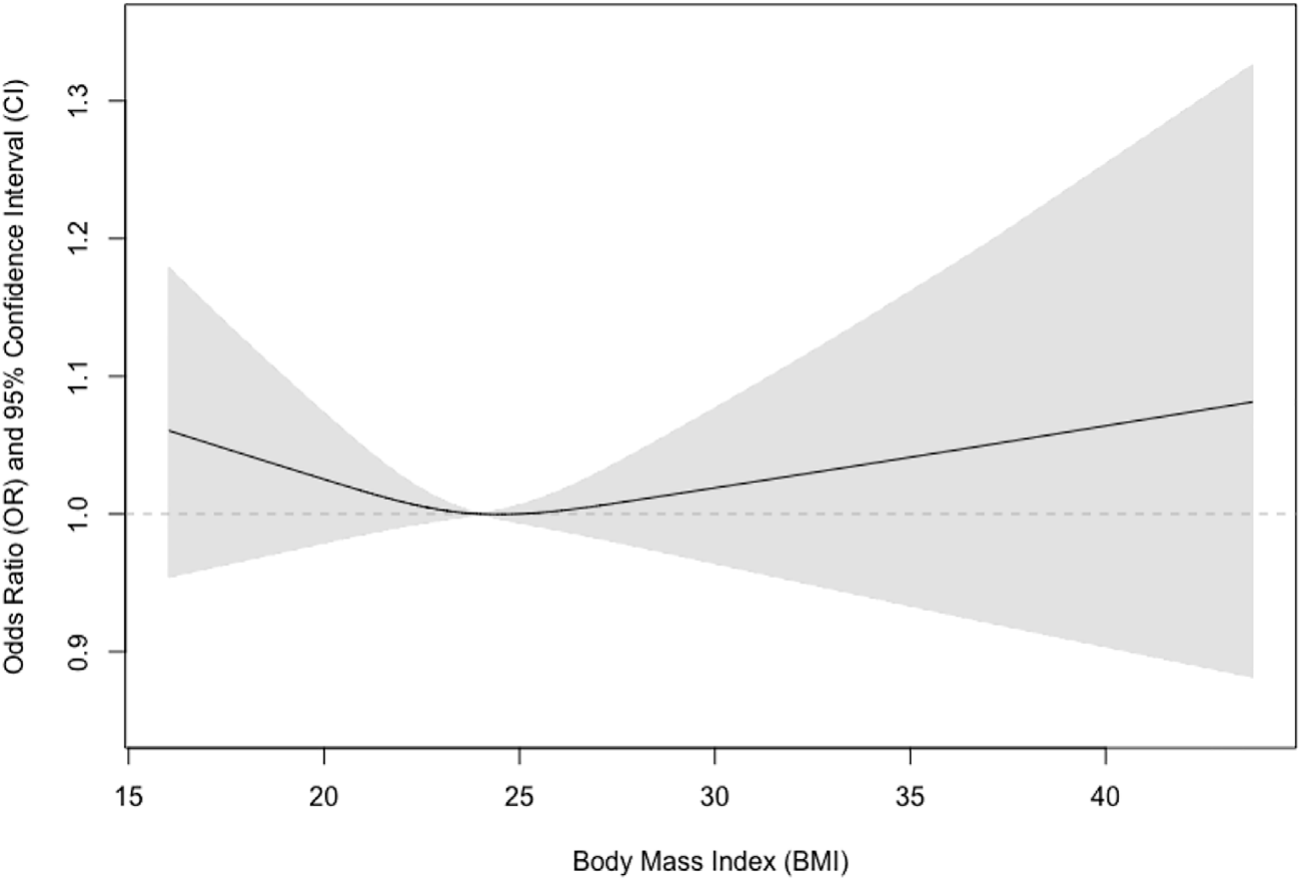
Association between body mass index and COPD in women.

In total, 126 participants could be classified as COPD and the prevalence of COPD was 10.4% (14.4% in men and 6.4% in women) in the study population, while that of COPD was 16.9 in current smokers and 7.2% in non-smokers. Among the 126 participants with COPD, 37 (29.4%), 71(56.3%), 12 (9.5%), and 6 (4.8%) were graded as mild, moderate, severe, and very severe according to the GOLD criteria. We performed additional analysis to examine the relationship between BMI and COPD and found similar patterns (Figures 4–6).

## Discussion

In the present study, we investigated the prevalence of pulmonary airflow limitation and its association with BMI in a community-based sample in China and found that the relationship between BMI and pulmonary airflow limitation was non-linear and sex-specific. A higher BMI was associated with lower risks of pulmonary airflow limitation in a linear fashion in men, while a U-shaped relationship was revealed in women.

Several previous studies reported the associations between obesity and pulmonary function or COPD in diverse populations. A recent large epidemiological survey of more than 200,000 Korean participants found that being underweight was associated with a higher risk of impaired pulmonary function (predicted FEV1% < 80%) in comparison with normal weight after taking into account a few potential confounders (Do et al., 2019). And a recent meta-analysis pooled thirty studies involving 1,578,449 participants also found that obesity had a higher risk of COPD with ORs (95% CIs) being 1.96 (95% CI: 1.78–2.17) for being underweight, 0.80 (95% CI: 0.73–0.87) for being overweight, and 0.86 (95% CI: 0.73–1.02) for being obese (Zhang et al., 2021). Further, another meta-analysis summarizing results from clinical trials found that the estimated rate of FEV_1_ decline decreased with increasing BMI, supporting that a higher BMI was associated with decreased risks of impaired pulmonary ventilation function (Sun et al., 2019). However, the results were not always consistent (Galesanu et al., 2014; Svartengren et al., 2020; Celli et al., 2021; Shin et al., 2022). For example, an earlier investigation in adolescents and adults found that there was a negative linear relationship between BMI and pulmonary function, implying that being overweight or obese was associated with higher risks of impaired pulmonary ventilation function (Davidson et al., 2014). The obesity paradox and discrepancies are not completely clear. One of the explanations may lie in the fact that some studies used data from the general population while others collected data only from patients with COPD. Further studies using longitudinal study design, repeated measurement of both obesity and pulmonary function as well as large samples are needed.

Our study has a few strengths. Firstly, we used validated spirometry to assess pulmonary function. The instrument has been validated and used in a couple of settings including epidemiological research. Secondly, we employed an interaction analysis to examine the potential effect modification effects of sex, and we found significant differences between men and women. Thirdly, we examined the potential non-linear relationships in the associations between BMI and the risks of impaired pulmonary ventilation function that were seldom investigated using restricted cubic splines. We found the relationship is almost linear in men and U-shaped in women.

A few limitations should also be acknowledged. Our survey employed a cross-sectional design. The built-in limitation of the cross-sectional study design implies that we cannot draw a firm causal conclusion for the associations between BMI and the risk of impaired pulmonary ventilation function. Longitudinal data with longer follow-up durations could strengthen our confidence in causal inference. Second, we are unable to employ a multistage sampling method to recruit participants because of the logistics and difficulties during the pandemic. The convenience sampling approach may underestimate the prevalence of impaired pulmonary ventilation function or COPD in this area. However, our sampling approach is much better than social media-based approaches which are commonly used during the pandemic nowadays. Moreover, it has been shown that obesity is a dynamic process and should be assessed repeatedly. Future research could incorporate both subjective and objective assessments of obesity levels.

In summary, our present analysis of this survey found that the relationship between BMI and pulmonary airflow limitation was U-shaped in women and linear in men. Future studies are warranted to clarify the biological mechanisms and shed light on these associations.

## Data Availability

The original contributions presented in the study are included in the article/Supplementary Material, further inquiries can be directed to the corresponding author.
